# Characterization and Application of Non-Formaldehyde Binder Based Citric Acid, Maleic Acid, and Molasses Adhesive for Plywood Composite

**DOI:** 10.3390/polym15193897

**Published:** 2023-09-27

**Authors:** Jajang Sutiawan, Alifah Syahfitri, Deni Purnomo, Sudarmanto, Narto, Fazhar Akbar, Dimas Triwibowo, Ismadi, Putri Amanda, Sukma Surya Kusumah, Muhammad Adly Rahandi Lubis, Dede Hermawan, Ignasia Maria Sulastiningsih, Arif Nuryawan, Luthfi Hakim

**Affiliations:** 1 Department of Forest Products, Faculty of Forestry, Universitas Sumatera Utara, Medan 20155, Indonesia; jajang.sutiawan@brin.go.id (J.S.); arif5@usu.ac.id (A.N.); 2 Research Center for Biomass and Bioproducts, National Research and Innovation Agency, Cibinong 16911, Indonesia; deni007@brin.go.id (D.P.); suda015@brin.go.id (S.); nart001@brin.go.id (N.); fazh001@brin.go.id (F.A.); dima018@brin.go.id (D.T.); isma011@brin.go.id (I.); putr011@brin.go.id (P.A.); sukm002@brin.go.id (S.S.K.); muha142@brin.go.id (M.A.R.L.); ignasiasulastin@gmail.com (I.M.S.); 3 Forest Product Department, Faculty of Forestry and Environment, IPB University, Bogor 16680, Indonesia; alifahsyahfitri@apps.ipb.ac.id (A.S.); dedehe@apps.ipb.ac.id (D.H.)

**Keywords:** eco-friendly composite, Indonesian wood, non-formaldehyde adhesive, plywood

## Abstract

Emissions of formaldehyde from wood-based panels, such as plywood, are gaining increased attention due to their carcinogenic impact on human health and detrimental effects on the environment. Plywood, which is primarily bound with a urea-formaldehyde adhesive, releases formaldehyde during hot pressing and gradually over time. Therefore, this study aims to analyze the impact of non-formaldehyde adhesive types on plywood performance. In addition, plywood performance was assessed by comparing Jabon wood (*Anthocephalus cadamba* Miq) veneer with other Indonesian wood veneers such as Mempisang (*Alphonse* spp.) and Mahogany (*Swietenia mahagoni*). To manufacture a three-layer plywood panel, a two-step manufacturing process was devised. The first step involved the use of Jabon veneers treated with citric acid (CA), maleic acid (MA), and molasses (MO), and another step was carried out for various wood veneers such as Jabon, Mempisang, and Mahogany using CA. The performance of plywood was examined using JAS 233:2003. The performance of plywood bonded with CA was better than that of plywood bonded with MA and MO. The Jabon wood veneer resulted in a lower density of plywood than other wood veneers. The water absorption, thickness swelling, modulus of elasticity, and tensile shear strength of plywood from Jabon wood veneer were similar to those of plywood from Mahogany wood veneer and lower than those of Mempisang wood veneer. The ester linkages of plywood bonded with CA were greater than those of plywood bonded with MA and MO because plywood bonded with CA has better performance than plywood bonded with MA and MO.

## 1. Introduction

The expansion of the home construction and furniture industries is driving global plywood production and consumption, which has experienced a significant increase from 158 million m^3^ to 162 million m^3^ [1]. Additionally, to meet the needs of importers such as Japan, the Republic of Korea, and the United States, Indonesia exports 3.85 million m^3^ of plywood annually [2]. Plywood can be used in various applications, including furniture, musical instruments, modes of transportation, packaging, sporting goods, and construction [3]. Notably, the manufacturing of plywood has witnessed a transformation in the use of fast-growing wood species. These species, which are prized for their short rotation cycles and suitable harvest diameters, have emerged as an alternative source for veneer-based products [4]. This shift signifies a sustainable method to meet the ever-expanding demand for plywood while mitigating the impact on natural wood forests.

Plywood is typically bonded with urea-formaldehyde (UF) adhesive due to its cost-effectiveness, rapid curing, transparent glue line, and ability to produce high-quality panels that meet the required standards [5]. Additionally, plywood adhesive such as phenol-formaldehyde (PF), melamine-urea-formaldehyde (MF), and polyurethane adhesive can be substituted [6,7]. However, the use of UF, MF, and PF resins in plywood manufacturing, both during the hot-pressing process and in the panel’s lifespan, poses environmental concerns and health risks [8] including conditions like nasopharyngeal cancer, leukaemia, respiratory tract irritation, genotoxicity, and skin sensitization [9,10,11,12]. Several effective techniques have been devised to mitigate formaldehyde emissions from wood-based panels joined with formaldehyde-based resins. [13,14,15,16,17,18,19]. Kristak et al. [8] reported that the emission of formaldehyde from wood-based panels joined with formaldehyde-based resins can be reduced using several techniques. The primary ones include (i) lowering the formaldehyde-urea molar ratio; (ii) changing the hot-pressing parameters, such as the temperature and duration used during pressing; (iii) including formaldehyde scavengers like tannins, lignin, starch, wheat flour, and rice husk flour; and (iv) the use of alternative adhesive systems based on carbon materials, modified amide-containing biopolymer, carboxymethyl cellulose, and soy flour, post-treatment of the wood-based goods, surface treatment, or combining UF resin with other resins. In addition, non-formaldehyde adhesives have been developed to reduce formaldehyde emissions [20]. Non-formaldehyde adhesives were successfully fabricated as wood adhesives [21,22,23,24,25,26,27]. The latter have been considered the cheapest non-formaldehyde adhesives (CA) with remarkable adhesion properties [28,29,30].

In 2012, a significant advancement emerged with the development of citric acid (CA) as a composite adhesive. Some of these developments include adhesive for molding [31,32,33], plywood [34,35,36,37], laminated veneer lumber [38], oriented strand board [39], composite plywood [40], and particleboard [24,41]. Notably, CA adhesive has gained widespread recognition and use in the domain of particleboard adhesives. Some of these developments include particleboard from bamboo [42,43], Nypa [44], Salacca [45], Imperata cylindrica [46], rice biomass [47], Washingtonia palm caches [48], giant reed [49], cardoon leaf [50], and rubberwood [51]. Sutiawan et al. [37] successfully used non-formaldehyde adhesives such as CA adhesive for Jabon (*Anthocephalus cadamba* Miq), a fast-growing species of plywood. The results showed that plywood pressed at 190 °C for 10 min had less delamination and higher tensile shear strength (TSS). Additionally, some properties of plywood complied with the requirements of JAS 233:2003. In this study, the Jabon and CA adhesive combination was compared to other Indonesian wood veneers and other non-formaldehyde adhesives such as maleic acid (MA) and molasses (MO).

Sutiawan et al. [52] reported that JIS A 5908-2003 [53] type 8 was satisfied by the performance of a sorghum bagasse composite particleboard bonded with MA. In addition, Sutiawan et al. [54] highlighted the application of MA in bonding table tennis blades fabricated from sorghum bagasse particleboard. The optimum condition of the previous study was an MA content of 15 wt%, particle size of 4–20 mesh, and pressing process conditions of 200 °C and 20 min [55]. Expanding on this investigation, Syahfitri et al. [56] achieved favorable outcomes by combining sorghum biomass with MO to produce particleboards. According to JIS A 5908:2003, the performance of the particleboard fulfilled the required standards. The optimum condition of the previous study was an MO content of 20 wt% and particle size of 4–20 mesh [56]. Therefore, this study aims to analyze the influence of non-formaldehyde adhesive on the performance of plywood. In addition, plywood performance was assessed by comparing Jabon wood veneer with other Indonesian wood veneers such as Mempisang (*Alphonse* spp.) and Mahogany (*Swietenia mahagoni*).

## 2. Material and Methods

### 2.1. Material

Three Indonesian wood veneers—Jabon, Mempisang, and Mahogany—of dimensions of 350 mm × 350 mm × 2 mm, were obtained from the Center for Standardization of Sustainable Forest Management Instruments, Bogor, Indonesia. The respective densities of the Jabon wood, Mempisang, and Mahogany used were 0.40 g/cm^3^, 0.67 g/cm^3^, 0.58 g/cm^3^. For consistency, the veneers were dried at 60 °C for 24 h, resulting in an MC of 5%, which was used throughout the study. The CA and MA were obtained from MERCK and PT Telagasakti Sakatautama, respectively. In addition, MO PTPN XII East Java, Indonesia provided the materials. According to previous investigations, the CA, MA, and MO were set at concentrations of 59 wt%, 44 wt%, and 59 wt%, respectively [37,52,56]. The adhesives used in this study have a solids content of 44.23–58.8%, gel time of 4.6–10.3 min, pH of 1.17–4.79, and viscosity of 6.5–152 mPa·s.

### 2.2. Characterization of Adhesive

The adhesive characteristics tested consisted of solids content, gelation time, pH, and viscosity.

#### 2.2.1. Solids Content

The solids content of the adhesive identifies the number of particles in the adhesive. The more adhesive particles that react with wood in the gluing process, the stronger the bond strength. An adhesive sample of 1 g was added on aluminum foil and then placed in an oven (Memmert, Germany) at 103 ± 3 °C for 3 h. After the sample dried, the aluminum foil was transferred to a desiccator and weighed. The solids content was calculated using the formula below:

Solids content (%) = (Oven-dried weight/Initial weight) × 100

#### 2.2.2. Gelation Time

To evaluate gelation time, the adhesive was placed in a test tube. The gel time meter (Techne GT-6, Coleparmer, Vernon, IL, USA) was positioned to submerge the needle in the sample. Dimethyl sulfoxide (DMSO) was used in a water bath, and the temperature was raised to 135 °C. After that, the time required for the adhesive to gelatinate was observed. The adhesive gelation time limit was obtained when the timer stopped automatically and showed the gelation time number marked “gel” on the screen.

#### 2.2.3. Viscosity

Approximately 20 mL of the adhesive samples were introduced into a glass and mounted on a rotational rheometer (RheolabQC, AntonPaar, Graz, Austria). Viscosity measurements were performed using a concentric cylinder (cc)-type spindle no. 27 with a rotation speed of 100/s. Tests were carried out at 25 °C to determine the viscosity, and dynamic viscosity was measured for 120 s.

#### 2.2.4. pH Value

The pH value of the adhesive was determined using a pH meter (Laqua pH 1200, Horiba, Kyoto, Japan). The pH value was shown on the screen a few moments after the electrode probe of the pH meter was dipped into the adhesive sample placed in a container.

#### 2.2.5. Curing Behaviors

The curing behaviors of the adhesive were examined using differential scanning calorimetry (DSC) and thermogravimetric analysis (TGA). The adhesive samples were analyzed using TGA, and all samples were freeze-dried for one hour and then pulverized to less than 60 mesh. TGA was conducted using a TGA 4000 instrument (PerkinElmer 4000, Waltham, MA, USA). The sample adhesive was analyzed using DSC. The samples were scanned from 25 °C to 500 °C at a 10 °C/min rate under nitrogen purging. DSC measurements were completed using a DSC 4000 instrument (PerkinElmer 4000, United States). Under nitrogen purging, the samples were scanned from 25 °C to 400 °C at 10 °C/min.

### 2.3. Manufacture of Plywood

A layer of 134 g/m^2^ based on the solids content of non-formaldehyde adhesive with a single glue line was spread on the wood veneer surface [38]. According to a previous study, a three-layer plywood panel was created using pressing conditions of 190 °C, for 10 min, under 1.3 MPa [37]. In this study, a two-step manufacturing process was devised (Table 1). In the first step, Jabon veneer was created using several non-formaldehydes adhesive such as CA, MA, and MO. Then, in the second step, another plywood panel was created using CA adhesive for various wood veneers such as Jabon, Mempisang, and Mahogany.

### 2.4. Determination of Plywood Performance

The performance of plywood, including density, moisture content (MC), water absorption (WA), thickness swelling (TS), modulus of elasticity (MOE), modulus of rupture (MOR), and TSS, was examined according to the Japanese Agricultural Standard No. 233 (JAS 2003) [57].

#### 2.4.1. Density

Density testing was carried out using samples measuring 5 × 5 × 0.6 cm^3^ in length, width, and thickness. The determination of density was expressed in the results of the comparison between the weight and volume of the board. The density of plywood was calculated using an equation based on the JAS 234-2003 standard [58].

Density (g/cm^3^) = M/V

where:

M is the weight of plywood (g)
V is the volume of plywood (cm^3^)

#### 2.4.2. Moisture Content (MC)

An MC test was performed on samples measuring 5 × 5 × 0.6 cm^3^ in length, width, and thickness. Subsequently, the MC value was calculated using the difference between the initial weight and the final weight after 24 h of drying in an oven at 103 °C. The MC value was calculated using an equation based on the JAS 234-2003 standard.

MC (%) = (BB − BKT)/BKT × 100

where:

BB is the weight of the sample before drying (g)
BKT is the weight of the sample after drying (g)

#### 2.4.3. Water Absorption (WA)

WA tests were performed on samples measuring 5 × 5 × 0.6 cm^3^. The difference in weight before soaking and weight after immersion in water for 24 h was measured. The WA value was derived using a calculation based on the JAS 234-2003 standard.

WA (%) = (B2 − B1)/ B1 × 100

where:

B1 is the weight of the sample before soaking (g)
B2 is the weight of the sample after soaking (g)

#### 2.4.4. Thickness Swelling (TS)

TS tests were performed on samples of 5 × 5 × 0.6 in length, width, and thickness. The measurements were carried out by measuring the difference in initial thickness before and after soaking for 24 h. The TS value was calculated using an equation based on the JAS 234-2003 standard.

TS (%) = (T2 − T1)/T1 × 100

where:

T1 is the thickness of the sample before soaking (mm)
T2 is the thickness of the sample after soaking (mm)

#### 2.4.5. Modulus of Elasticity (MOE) and Modulus of Rupture (MOR)

MOE and MOR tests were performed on samples measuring 20 × 5 × 0.6 cm^3^. A universal testing machine (Shimadzu AG-IS 50 kN, Japan) was then used to test the results. MOE and rupture tests were performed at 10 mm/minute loading speeds. The MOE and MOR were determined using an equation based on the JAS 234-2003 standard.

MOE (MPa) = (∆PL3)/(4∆Ybh)

MOR (MPa) = (3PmaxL)/(2bh)

where:

P max is the maximum load (N)
P is the load below the limit of proportion (N)
Y is the deflection at load P (mm)
L is the pacing distance (mm)
b is the width of the test sample (mm)
h is the thickness of the test sample (mm)

#### 2.4.6. Tensile Shear Strength (TSS)

The test was carried out using samples measuring 8 × 2.5 × 0.6 cm^3^. The samples were sheared using a universal testing machine (Shimadzu AG-IS 50 kN, Kyoto, Japan) at a loading speed of 2 mm/min to a maximum load. Adhesive strength was calculated using the JAS 234-2003 standard.

Shear strength (MPa) = P/(b × h)

where:

P is the maximum load (N),
b is the width of the specimen (mm)
h is the distance between notches (mm)

### 2.5. Functional Groups Analysis

A Fourier transform infrared (FTIR) instrument was used to measure changes in functional groups (PerkinElmer, USA). An FTIR spectra range from 4000 to 400 with a 4 cm^−1^ resolution was captured in absorbance mode. The spectra were normalized for baseline using Perkin Elmer software (Spectrum, Version 10.5.1) [52].

### 2.6. Statistical Analysis

A simple completely randomized design was employed, featuring two factors: the type of non-formaldehyde adhesive (CA, MA, and MO) and the type of wood veneer (Jabon, Mempisang, and Mahogany). The difference in plywood properties was analyzed using analysis of variance (ANOVA) and Duncan’s multi-range test (Duncan) at 0.05.

## 3. Result and Discussion

### 3.1. Characteristics of Adhesive

**Figure 1:**
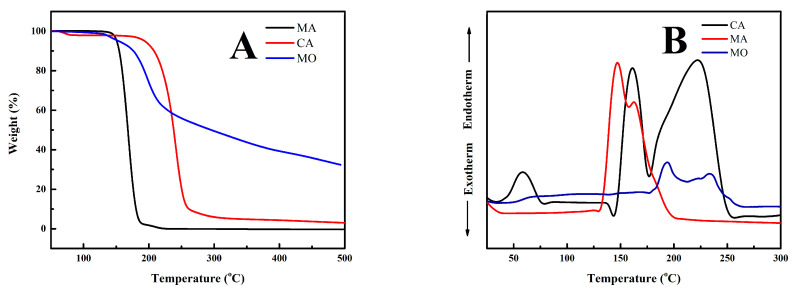
Thermal characteristics of adhesives used in this study (**A**) TGA and (**B**) DSC.

The results regarding adhesive characterization (refer to Table 2) reveal that the MA adhesive has lower solids content, pH, and viscosity than the CA and MO adhesives. The lower pH and viscosity contributed to the lower quality of plywood bonded with MA adhesive. The short gelation time indicates that the adhesive no longer required a long setting time during hot pressing in the manufacture of composite products [59]. The average viscosity of the CA, MA, and MO adhesives is quite low compared to conventional formaldehyde-based plywood adhesives, such as UF resins with an average viscosity of around 250–400 mPa·s [59]. The viscosity value affects the ability of the adhesive to penetrate the pores of the wood and the storage life of the adhesive. Adhesives with high viscosity have a short storage life because they harden faster and the quality of the adhesive is low [59]. Notably, the MA and MO adhesives have lower thermal degradation (145 °C and 150 °C) compared to the CA adhesive (165 °C) (Figure 1A). However, the MO adhesive also has a higher first endothermic peak (200 °C) compared to the MA and CA adhesives (140 °C and 159 °C) (Figure 1B). These phenomena resulted in low TSS in plywood bonded with MO adhesive.

### 3.2. Variation in Type of Non-Formaldehyde Adhesive

**Figure 2:**
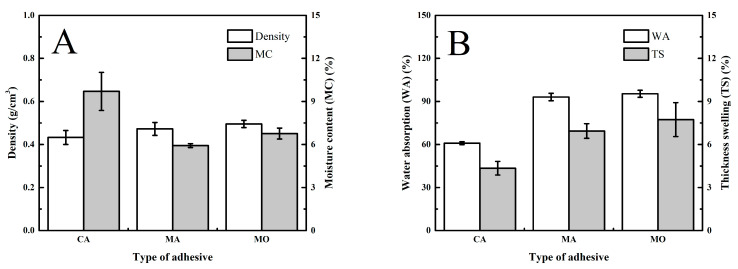

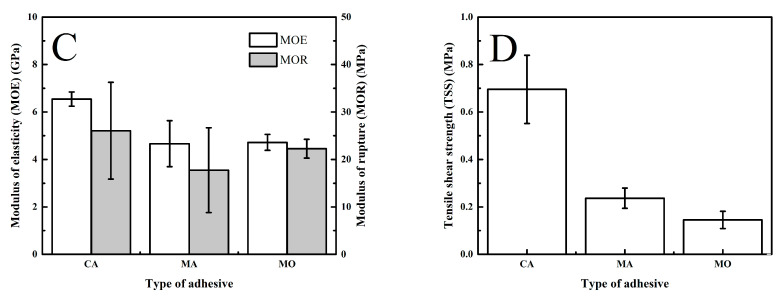
Influence of non-formaldehyde adhesive type on the properties of plywood. Note: error bars represent the standard deviation. (**A**) Density and moisture content, (**B**) water absorption and thickness swelling, (**C**) modulus of elasticity and rupture, and (**D**) Tensile shear strength.

Figure 2 shows the influence of non-formaldehyde adhesive type on the performance of plywood observed using Jabon plywood. The density and MC of plywood ranged between 0.43 and 0.50 g/cm^3^ and between 5.92 and 9.70%, respectively (Figure 2A). The ANOVA revealed that there was no significant difference between plywood densities (Table 3). However, the MC of plywood bonded with CA was slightly higher than that of plywood bonded with MA and MO (Table 4). The samples’ WA and TS ranged from 60.93 to 95.39% and from 4.35 to 7.74%, respectively (Figure 2B). The WA of plywood bonded with MO was slightly higher than that of plywood bonded with CA and MA. This phenomenon was affected by a cross-linker in CA and MA, and lignocellulose material was higher than in MO. Sutiawan et al. [37] reported that CA adhesive resulted in a higher concentration of cross-linkers in ester linkage than other adhesives, as detected by FTIR analysis.

As shown in Table 4, the MOE and MOR of plywood bonded with CA were marginally greater than in plywood bonded with MA and MO at a *p*-value of 0.05. The TSS of plywood bonded with CA was marginally greater than that of plywood bonded with MA and MO at the same level of *p*-value, which was the same as the MOE and MOR (Table 4). According to previous studies, these occurrences are caused by the presence of hydroxyl groups (OH), which are necessary for reacting with carboxyl groups (COOH) of CA to produce ester linkages (R-COO-R) [52].

### 3.3. Variation of Wood Veneer

**Figure 3:**
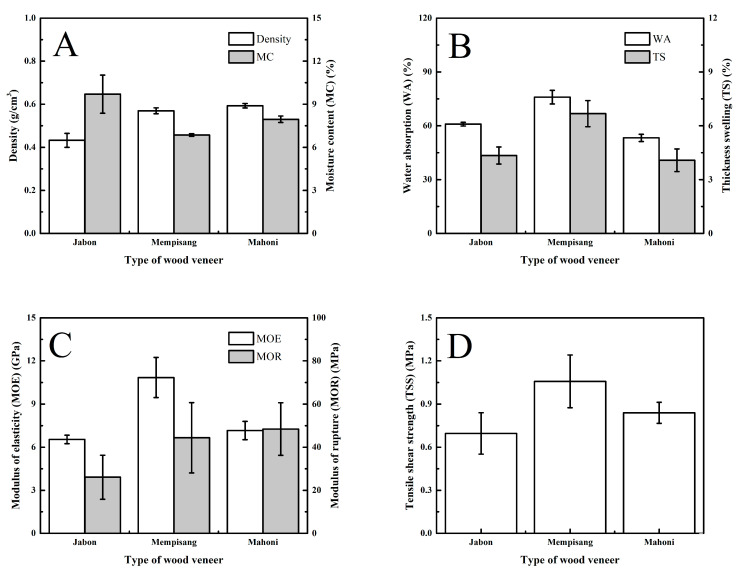
Influence of type of wood veneer on the performance of plywood. Note: error bars represent the standard deviation. (**A**) Density and moisture content, (**B**) water absorption and thickness swelling, (**C**) modulus of elasticity and rupture, and (**D**) Tensile shear strength.

The impact of wood veneer type on plywood performance was observed using CA adhesive, as illustrated in Figure 3. The average density of plywood ranged from 0.43 to 0.59 g/cm^3^ and the MC ranged from 6.85 to 9.70%, as shown in Figure 3A. The Jabon wood veneer has a lower density of plywood than other wood veneers (*p* < 0.05, Table 5 and Table 6). These phenomena are due to the density of Jabon wood (0.40 g/cm^3^) being lower than that of Mempisang (0.67 g/cm^3^) and Mahogany (0.58 g/cm^3^) [60,61,62]. Karliati et al. [60] reported that the manufacture of wood-derived products, such as plywood, could increase the product’s density compared to solid wood. According to the JAS 233: 2003 standard (JAS 2003), the MC of all plywood samples was inappropriate according to the standard (MC < 14%).

The WA and TS of plywood from Jabon wood veneer (60.93% and 4.35%) were similar to those from Mahogany wood veneer (53.27% and 4.08%) and were lower than those from Mempisang (75.97% and 6.68%) wood veneer (*p* < 0.05, Table 5 and Table 6) (Figure 3B). A previous study reported that Jabon plywood bonded with PF adhesive has a WA of 107.6% [60]. Therefore, the plywood in this current study showcases commendable dimensional stability. The formation of ester linkages in wood-derived products has resulted in good dimensional stability [24,43,63].

The MOE and MOR of plywood from Jabon wood veneer (6.54 GPa and 26.06 MPa) were lower than those from Mahogany (7.16 GPa and 48.42 MPa) wood veneer and Mempisang (10.85 GPa and 44.37 MPa) wood veneer (*p* < 0.05, Table 5 and Table 6) (Figure 3C). However, the TSS of plywood made with Jabon wood veneer (0.70 MPa) was similar to that of Mahogany wood veneer (0.84 MPa) and was lower than that of Mempisang (1.06 MPa) wood veneer (*p* < 0.05, Table 5 and Table 6) (Figure 3C,D). The density of wood-derived products affected mechanical properties such as bending and bonding quality [24]. As an example, Sutrisno et al. [64] reported that wood-derived products, specifically laminated veneer lumber bonded with PF resins, exhibited an MOR of 43.14 MPa, which surpasses the MOR value observed in this study. In addition, Sun et al. [34] reported that Poplar plywood bonded with CA has a TSS value of 0.35 MPa, making all TSS values higher.

### 3.4. Functional Groups Analysis

**Figure 4:**
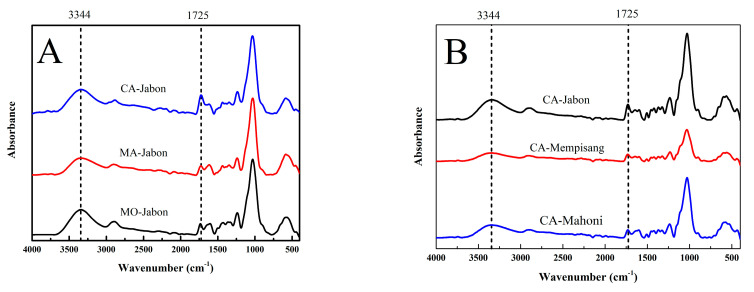
FTIR spectrum of plywood with different types of non-formaldehyde adhesive (**A**) and wood veneer (**B**).

The FTIR spectrum of plywood using different types of non-formaldehyde adhesive and wood veneers is shown in Figure 4. The difference is visible at approximately 1725 cm^−1^. The peak of plywood bonded with CA, approximately 1725 cm^−1^ (ester linkages), was greater (Figure 4A) than that of plywood bonded with MA and MO [52]. This distinction in peaks contributes to the superior performance of CA-bonded plywood when contrasted with MA- and MO-bonded plywood. In addition, the peaks at 1725 cm^−1^ (ester linkages) and 1040 cm^−1^ (hemiacetal’s C-O-C) in plywood from Jabon wood veneer were higher than those from Mahogany and Mempisang wood veneer (Figure 4B). This observation explains the similarity in TSS between plywood from Jabon wood veneer and plywood from Mahogany wood veneer, despite the lower density of Jabon wood. The peak at 1725 cm^−1^ signifies ester linkages, and the peak at 1040 cm^−1^ corresponds to hemiacetal’s C-O-C stretching vibration, resulting in the esterification of CA with lignocellulose materials [63,65].

## 4. Conclusions

In conclusion, the type of non-formaldehyde adhesive and the type of wood veneer affected the performance of plywood. Plywood bonded with CA showed superior performance compared to plywood bonded with MA and MO adhesives. The Jabon wood veneer resulted in a lower density of plywood than other wood veneers. The water absorption, thickness swelling, modulus of elasticity, and tensile shear strength of plywood crafted from Jabon wood veneer showed similarities to plywood made from Mahogany wood veneer and were lower than those derived from Mempisang wood veneer. The ester linkages of plywood bonded with CA were greater than those of plywood bonded with MA and MO because plywood bonded with CA has better performance than plywood bonded with MA and MO. Further study is still needed to determine solid content and hardener optimization factors when applying CA, MA, and MO in adhesive plywood.

## Figures and Tables

**Table 1:** Conditions of manufacture of plywood.

Steps	Type of Adhesive	Type of Wood Veneer
1	CA	Jabon
	MA	Jabon
	MO	Jabon
2	CA	Jabon
	CA	Mempisang
	CA	Mahogany

**Table 2:** Characteristics of adhesives used in this study.

Type of Adhesive	Solids Content (%)	Gel Time (min)	pH	Viscosity (mPa·s)
CA	56.20	4.6	2.3	7.3
MA	44.23	10.3	1.17	6.5
MO	58.8	5.9	4.79	152

**Table 3:** ANOVA of plywood properties with variation by type of non-formaldehyde adhesive.

Parameter	ANOVA
MOE	0.015 **
MOR	0.497 ^ns^
TSS	0.001 **
Density	0.084 ns
MC	0.003 **
WA	0.000 **
TS	0.005 **

^ns^ not difference, ** difference.

**Table 4:** Duncan test results of plywood properties with variation by type of non-formaldehyde adhesive.

Type of Adhesive	Density	MC	WA	TS	MOE	MOR	TSS
CA	0.43 a	9.70 c	60.93 a	4.35 a	6.54 b	26.06 a	0.70 b
MA	0.47 ab	5.92 a	93.08 b	6.94 b	4.66 a	17.74 a	0.24 a
MO	0.50 b	6.76 b	95.39 b	7.74 b	4.72 a	22.26 a	0.15 a

There is no difference between values with the same letter in a row.

**Table 5:** ANOVA of plywood properties with variation by type of wood veneer.

Parameter	ANOVA
MOE	0.002 **
MOR	0.166 ^ns^
TSS	0.051 ^ns^
Density	0.000 **
MC	0.012 **
WA	0.000 **
TS	0.004 **

^ns^ not difference, ** difference.

**Table 6:** Duncan test results of plywood properties with variation by type of adhesive.

Type of Wood Veneer	Density	MC	WA	TS	MOE	MOR	TSS
Jabon	0.43 a	9.70 b	60.93 b	4.35 a	6.54 a	26.06 a	0.70 a
Mempisang	0.57 b	6.85 a	75.97 c	6.68 b	10.85 b	44.37 a	1.06 b
Mahogany	0.59 b	7.95 a	53.27 a	4.08 a	7.16 a	48.42 a	0.84 ab

There is no difference between values with the same letter in a row.

## Data Availability

The data presented in this study are available on request from the corresponding author.
